# Prognostic Factors for Quality of Life After Interdisciplinary Pain Rehabilitation in Patients with Chronic Pain—A Systematic Review

**DOI:** 10.1093/pm/pnac098

**Published:** 2022-06-23

**Authors:** Seraina Liechti, Elena Tseli, Jan Taeymans, Wilhelmus Grooten

**Affiliations:** Department of Health Professions, Division of Physiotherapy, Bern University of Applied Sciences, Bern, Switzerland; School of Health and Welfare, Dalarna University, Falun, Sweden; Department of Neurobiology, Care Sciences and Society, Division of physical therapy, Karolinska Institutet, Huddinge, Sweden; Department of Health Professions, Division of Physiotherapy, Bern University of Applied Sciences, Bern, Switzerland; Department of Movement and Sport Science & Rehabilitation, Vrije Universiteit Brussel; Department of Neurobiology, Care Sciences and Society, Division of physical therapy, Karolinska Institutet, Huddinge, Sweden; Women’s Health and Allied Health Professionals Theme, Medical Unit Occupational Therapy and Physiotherapy, Karolinska University Hospital, Stockholm, Sweden

**Keywords:** Chronic Musculoskeletal Pain, Interdisciplinary Rehabilitation, Health-Related Quality of Life, Prognostic Factors, Systematic Review

## Abstract

**Background:**

Health-related quality of life (hrQoL) is a core outcome in evaluating interdisciplinary pain rehabilitation (IPR). This systematic review aimed to identify prognostic factors for hrQoL at least six months after IPR in chronic pain patients.

**Methods:**

A systematic search was conducted in MEDLINE, PsycINFO, EMBASE, CINAHL, Web of Science and Cochrane CENTRAL until September 2020. Included were prognostic studies on the outcome hrQoL in adults aged 18 to 67 years with chronic pain (excluding malignancies, systemic-, inflammatory or degenerative joint diseases) who had undergone IPR. Studies were assessed with The Quality in Prognostic Studies-tool. Potential prognostic factors at baseline for the domains pain, psychological and physical functioning were qualitatively synthesized for hrQoL. Grading of Recommendations Assessment, Development and Evaluation was used to evaluate the level of evidence.

**Results:**

Fourteen studies on 6,668 participants (mean age 37.4–52.8 y), with musculoskeletal pain/fibromyalgia and a pain duration ranging between 13.1 and 177.4 months were considered eligible. With a very low certainty of evidence, pain intensity, emotional distress, and physical functioning at baseline were inconsistent for prediction of hrQoL and pain duration was not predictive. With low certainty of evidence, fewer pain sites, lower levels of negative cognitive behavioral factors, and higher levels of positive cognitive behavioral factors predicted a better outcome.

**Conclusions:**

The overall certainty of evidence was low to very low, making it difficult to reach definitive conclusions at present. Future studies with a predefined core set of predictors investigating hrQoL in patients with chronic pain after IPR are needed.

## Introduction

The International Association for the Study of Pain (IASP) defined chronic pain, such as chronic musculoskeletal pain, chronic widespread pain or fibromyalgia, as pain in one or more anatomic regions that persists or recurs for more than 3 months; is associated with significant emotional distress and/or significant functional disability and the symptoms are not better explained by another diagnosis [1]. Worldwide, approximately 20% of adults suffer from pain, and about 10% are newly diagnosed with chronic pain each year [2]. Around 20% of the population in Europe is affected by chronic pain [3]. Chronic pain is a long-term condition affecting different aspects of patient’s health such as daily activities, mental health, sleep, cognitive processes, cardiovascular health and overall quality of life [4, 5]. To treat this long-term multifaceted condition a multimodal treatment approach is often essential [1, 6]. Interdisciplinary pain rehabilitation (IPR), in line with the definition of interdisciplinary treatment: “Multimodal treatment provided by a multidisciplinary team collaborating in assessment and treatment using a shared biopsychosocial model and goals” [7], is presently considered best evidence practice for this patient group [6, 8]. IPR includes different health care providers such as physician, physical therapist, psychologist, occupational therapist, or social worker. Applying a biopsychosocial approach to pain, the key components are physical activity/exercise, education, coping skills training, occupational therapy and pharmaceutical management, if needed [6, 8, 9]. The primary aim of IPR extends beyond pain reduction solely, and instead focuses on general improvements in physical, psychological and social aspects according to the patient's experience [10]. Evidence suggests positive effects of IPR on several outcomes however with small to moderate effect sizes [8, 9]. When assessing pain management trials, health-related quality of life (hrQoL) is recommended as an outcome [11] and an expert consensus statement lists hrQoL as one of the core outcomes to be measured in trials assessing IPR [10]. However, not all patients respond to the intervention and future research should focus on patient variables to define subgroups which would profit the most from IPR, identifying the need for prognostic research [9]. Prognostic research seeks to examine and predict future outcomes in people with a given disease or health condition. Well-conducted prognostic research helps to define those subgroups and is important for clinical decision making [12, 13]. Based on available data from pre-treatment assessments, clinicians may get an early indication of their patients prognosis, which can be subsequently targetet during the IPR.

Previous systematic reviews identified prognostic factors for patients suffering from pain in primary care [14, 15] or focused on predictive issues in patients with acute low back pain [16]. Other reviews exist that examine prognostic factors in fibromyalgia patients [17] or examine prognostic factors in chronic pain patients for outcome other than hrQoL [18, 19]. However, to the authors' knowledge, no systematic review or meta-analysis has been conducted on prognostic factors for hrQoL after IPR in patients with chronic pain.

The aim of this systematic review was to evaluate and meta-analyze published data on prognostic factors for hrQoL at least six months after IPR in patients with chronic pain.

## Methods

This systematic review with planned meta-analysis was conducted according to the Preferred Reporting Items for Systematic Reviews and Meta-Analyses (PRISMA) guidelines [20] and adapted to the guidelines from Riley et al. for systematic review and meta-analysis of prognostic factor studies [13].

A protocol was registered a priori with PROSPERO, Centre for Reviews and Dissemination, University of York, CRD-register (registration number: CRD42020195885).

### Eligibility Criteria

Eligibility criteria were defined with the modified PICOTS system, which frames all important parts of the research question and is helpful at different stages of the process: e.g., study identification and selection, and adapted for reviewing prognostic factor studies [13]; P (population), I (index prognostic factor), C (comparator prognostic factor), O (outcome), T (timing), and S (setting).

P: The population of interest were adults aged 18 to 67 years with chronic pain who had undergone an IPR. Chronic pain was defined as musculoskeletal pain with a duration of more than 3 months referred to as chronic primary pain and chronic secondary musculoskeletal pain mostly [1] and included common nonspecific pain such as back pain, neck pain, and generalized pain syndromes (i.e., fibromyalgia). Pain syndromes caused by malignancies, systemic or inflammatory diseases (i.e., rheumatoid arthritis) or degenerative joint diseases were exclusion criteria, i.e., diagnosis where there is a clear association between pain and disease. The IPR of the included studies followed the biopsychosocial model and was coordinated by minimal three different health professionals. The intervention included a physical component and either a psychological component or a social/work component or both [8].

“I” refers to index prognostic factors. These include any independent variable at baseline investigated for their potential to predict the outcome hrQoL at follow-up.

C: No comparator prognostic factor is being considered for this review.

O: The chosen outcome hrQoL is one of the core outcomes for effectiveness studies in IPR [10] and the selected patient reported outcome measures for hrQoL in this review have good psychometric properties for chronic pain patients [21–23].

T: Included studies investigated prognostic factors, measured at baseline, for the outcome hrQoL at follow-up at least 6 months post-intervention. Therefore, having a longitudinal design, either observational or experimental/clinical trials was an inclusion criterion.

S: The setting of the included studies is an IPR in which the prognostic factors at the beginning of the pain rehabilitation are examined for their influence on the outcome hrQoL. The treatment could be provided in an inpatient or outpatient setting and there was no specification for the duration or intensity of the treatment.

Included studies needed to be original research papers published in full-text and in peer-reviewed journals, and there was no language restriction.

### Data Sources and Search Procedure

To identify relevant studies, the following six electronic databases were searched: MEDLINE (Ovid), PsycINFO (Ovid), EMBASE (Elsevier), CINAHL (EBSCO), Web of Science (Clarivate Analytics plc), and the Cochrane Central Registry of Controlled Trials (CENTRAL). The search algorithms were created with the help of a professional librarian and are presented in the Supplementary Data (Table S1). The research was conducted in September 2020 and focused on studies published from 2000 on to this date. In addition, a manual search and a check of reference lists targeted other relevant articles. In case of unavailability, corresponding authors were asked to provide the full text.

### Study Selection and Quality Assessment

All studies found in the databases were downloaded and organized using Endnote Software [24] and duplicates were deleted. The study selection procedure was performed using the web application Rayyan [25]. All studies were screened in terms of title and abstract by two researchers (S.L., E.T.) independently. Any disagreements between reviewers were discussed and resolved by consensus. The remaining studies were screened for inclusion criteria by reading the full texts by the same two researchers independently. Any disagreements between reviewers were discussed and resolved by consensus and a third researcher (W.G.) was asked if no consensus could be found. The reasons for exclusion are given in accordance with the PICOT order. Hence, if a study can be excluded based on population and outcome, the reason is assigned to the population. The interrater agreement throughout the review process was evaluated calculating Cohen’s kappa [26].

The included studies were assessed for internal validity with The Quality in Prognostic Studies (QUIPS)-tool, which is a Cochrane-based tool for evaluating validity and bias in studies of prognostic factors [27]. QUIPS appraises the risk of bias (RoB) in the following six domains: study participation, study attrition, prognostic factor measurement, outcome measurement, study confounding, and statistical analysis and reporting.

For each included study, the six domains were rated as high, moderate, or low RoB by two researchers (S.L., W.G.) independently and disagreements were discussed to find a consensus. A threshold for the levels of RoB was set a priori for some characteristics, that is, permitted participation and attrition rate, according to previous recommendations for a pain rehabilitation perspective as derived from our research group [28]. The overall RoB for each study was evaluated as follows: A study was classified to have high RoB when one or more domains had high RoB or when three or more domains were rated as moderate RoB. A moderate RoB consisted of a maximum of two domains with moderate RoB and the rest low RoB. When at least five of the domains had a low RoB and none of the domains had a high RoB the study was rated as low RoB [18, 28].

### Data Extraction

Data extraction was guided by the adapted checklist for critical appraisal and data extraction for systematic reviews of prediction modelling studies (CHARMS-PF) checklist for primary studies of prognostic factors [13]. Data on the country of origin, source of data, population, intervention characteristics, outcome measures regarding hrQoL, potential prognostic factors and assessment methods and statistical analyses were extracted by one researcher (S.L.) into MS Excel-tables [29] and checked by another researcher (E.T.). Only the data regarding the outcome hrQoL and the chosen domains of prognostic factors at baseline were extracted. Data needed for statistical analyses were imported in an extra SPSS file for further analysis. Corresponding authors were contacted through e-mail for missing data or additional details if needed.

### Data and Evidence Synthesis

A narrative synthesis of the included studies was performed to present the direction of the association between the prognostic factors at baseline and the outcome hrQoL. A statistically significant association with the outcome was defined as a univariate association or an association adjusted for confounders or other prognostic variables, with a *P* values < .05 and was taken as evidence for the factor’s potential prognostic value. The association was classified as either positive, negative or absent. For all statistically non-significant prognostic factors, the direction was recorded if reported. In the conceptualization of potential prognostic factors, we followed the study by Tseli et al. and organized the factors into the following domains [18]: pain-related factors, physical functioning-related factors, psychological factors, sociodemographic-related and medical-related factors. Due to a large amount of data in this review the prognostic factors were narrowed down to pain, psychological factors and physical functioning-related factors. Pain factors were later grouped into three subgroups: pain intensity, pain duration, and pain sites, as were psychological factors which were sorted into: emotional distress, for factors such as depression, and cognitive-behavioral negative or positive factors. Cognitive behavioral factors were grouped into positive factors such as self-efficacy and optimism and negative factors such as catastrophizing to order to obtain a logical direction of association before the synthesis. For each prognostic factor, the direction of the association and the significance level were tabulated, and the results were interpreted as “inconsistent” on domain level if there were statistically significant associations in both directions. Furthermore, subgroup analyses were done by exploring whether the statistically significant associations and directions were affected by subgrouping the different outcome domains of hrQoL, the different overall RoB, the different follow-up time or the study sample.

The Grading of Recommendations Assessment, Development and Evaluation method (GRADE) tool adapted for reviews of prognostic studies was used to rate the certainty (quality) of scientific evidence in this systematic review [30]. In the original GRADE, the highest certainty of evidence for effectiveness is based on randomized controlled trials (RCT), while in the present study focusing on prognosis, cohort studies are the prime study design. The prognostic factor studies were rated based on phase of investigation which refer to the robustness of the predictive value. The certainty of evidence was thereby classified into high, moderate, low, or very low certainty of evidence by discussion among three researchers (S.L., E.T., W.G.).

## Results

### Study Selection

**Figure 1.**
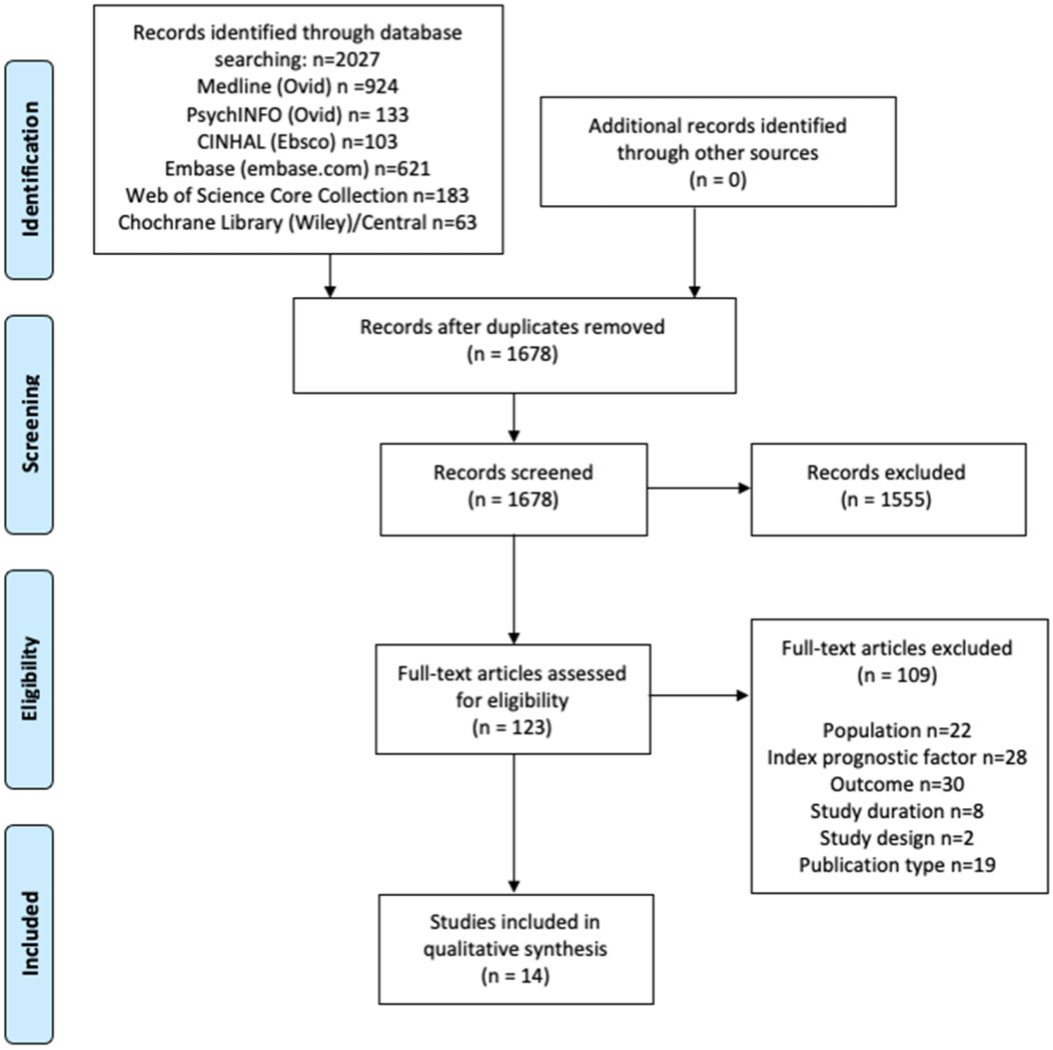
PRISMA flow diagram.

A total of 2027 records were found (Medline n = 924, PsychINFO n = 133, CINHAL n = 103, Embase n = 621, Web of Science Core Collection n = 183, Cochrane Library (Wiley)/Central n = 63). After elimination for duplicates in Endnote, 1678 studies remained for further evaluation. Titles and abstracts were screened on the inclusion and exclusion criteria and 123 studies were read in full text for PICOT eligibility. Fourteen studies remained after screening these full texts and were included for synthesis in this review (Figure 1). The interrater agreement throughout the review process for title/abstract screening and full text screening showed a moderate level of agreement (0.62 and 0.63 Cohen’s kappa, respectively) [26].

### Description of the Included Studies

The 14 included studies were published between 2007 and 2020 and were all conducted in Europe (Table 1): 10 cohort studies [31–40], one randomized control trial [41], and three register studies [42–44]. Study sample size ranged from 54 [41] to 2,876 [44], totaling 6,668 patients. In one study [41], only the data of a subgroup was analyzed, and one study [37] only examined the 100 patients with the greatest improvement (responders) and the 100 patients with the largest decrease (non-responders) in hrQoL. The average age of the patients ranged from 37.4 [31] to 52.5 years [37]. The percentage of females in the different study samples ranged from 52.8% [35] to 100% [36]. The population diagnoses of the included studies were described as chronic pain [32, 44], chronic low back pain [33–35], chronic non-malignant pain [37, 42], chronic musculoskeletal pain [39, 40, 43], fibromyalgia [36, 38, 41] and Whiplash associated disorder/chronic neck pain [31]. The patients’ average pain duration ranged from 13.3 [31] to 177.4 [41] months and the follow-up period varied between six [31–36, 39, 41] and 12 months [37, 38, 40, 42–44].

**Table 1:** Description of population and intervention

Author (year)	Country	Source of Data	Population	Intervention
Angst et al. (2014)	Switzerland	Cohort study	D: Whiplash associated disorder, chronic neck pain n: 175 age: 37.4 (11.7) female: 79.4% duration: 13.3 (10.7) mo	*Intervention profile*: interdisciplinary program *Intervention time*: 4 w *Details*: individual physiotherapy and physiotherapy in small groups, medical training therapy (graded exercise), passive therapy modules, occupational therapy, creative therapy, neuropsychological treatment with group information about pain, individualized CBT *Setting*: inpatient
Bremander et al. (2011)	Sweden	Cohort study	D: chronic pain n: 97 age: 44.6 (9.7) female: 88% duration: ≥3 months	*Intervention profile*: multidisciplinary rehabilitation program *Intervention time*: 3 w inpatient and 6 mo outpatient care *Details: g*roup discussions with a cognitive approach and a bio-psychosocial perspective on pain, body awareness therapy, pool exercise, qi-gong and individual counselling *Setting*: outpatient
Buchner et al. (2007)	Germany	Cohort study	D: chronic low back pain n: 387 age: 44.1 (range 18–65) female: 57.6% duration (subgroups): stage I and II: 9.6 (1.2) mo stage III: 11.9 (10.9) mo stage IV: 26.6 (20.6) mo	*Intervention profile*: multidisciplinary treatment *Intervention time*: 3 w, total of 120 h, 8-hour sessions, 5 d/w *Details*: individual therapy: physical exercises, ergonomic training, psychotherapy (analysis of psychosocial factors, explaining nature and function of their pain, behavior therapy, stress relaxation), patient education (regarding pain, physical and mental coping strategies, work, lifestyle), behavioral therapy and workplace-based interventions; group therapy: CBT, workout exercises, relaxation training, work-related training, individually tailor medical training therapy and exercises under the direction of a physiotherapist *Setting*: inpatient
Dong et al. (2019)	Sweden	Cohort register based (SQRP)	D: chronic non-malignant pain n: 872 age: 45.8 (10.5) female: 80.3% duration (median, range in mo): normal weight: 57.7 (27–136.3) overweight: 64.7 (30.3–162.7) mild obesity: 91.1 (30–220.5) severe obesity: 123.4 (36.4– 238.5)	*Intervention profile*: interdisciplinary multimodal pain rehabilitation *Intervention time*: on average 10 w *Details*: pain education, supervised physical activity, training in simulated environments, CBT *Setting*: outpatient
Farin et al. (2013)	Germany	Cohort study	D: chronic low back pain n: 688 age: 51 (11.2) female: 57.2% duration: <1 year: 13% 1–2 years: 11.1% 3–5 years: 18.6% 6–10 years: 16.3% >10 years: 40.2%	*Intervention profile*: multidisciplinary pain treatment *Intervention time*: 3 w with a mean length of 20.6 days, generally 4–5 therapy sessions a day on workdays. *Details*: educational, somatic, psychotherapeutic, social and occupation-related therapy with the following treatment elements: information (e.g. on chronic back pain), training based on a bio-psychosocial disease model, occupational therapy, physical therapy, exercise therapy, psychotherapeutic treatment to modify mal-adaptive illness behavior, relaxing techniques and coping with stress *Setting*: in and outpatient
Gerdle et al. (2016)	Sweden	Cohort register based (SQRP)	D: chronic musculoskeletal pain n: 227 age: 38.1 (10.1) female: 81.6% duration: 83.8 (85.8) mo	*Intervention profile*: interdisciplinary multimodal pain rehabilitation *Intervention time*: 6–8 w for at least 20 h per w *Details*: groups session: physiotherapy, ergonomics, training in coping strategies, education in pain management, work related advice and support, individually tailored sessions with team members *Setting*: outpatient
Glattacker et al. (2010)	Germany	Cohort study	D: fibromyalgia n: 245 age: 52.0 (9.0) female: 100% duration: <2 years: n = 28; 11.4% 3–5 years: n = 38; 15.5% 6–10 years: n = 62; 25.3% >10 years: n = 114; 46.5%	*Intervention profile*: multimodal therapy *Intervention time*: 3 w *Details*: patient education concerning pain management as well as drugs, physiotherapy (including nordic walking and exercise), physical therapy, psychological treatment (autogenic training, muscle relaxation, coping with pain) in groups or in individual therapy if necessary *Setting*: inpatient
Glattacker et al. (2018)	Germany	Cohort study	D: chronic LBP n: 214 age: 50.7 (10.2) female: 52.8% duration: <1 year: n = 22; 10.3% 1–2 years: n = 20; 9.2% 3–5 years: n = 33; 15.4% 6–10 years: n = 32; 15% >10 years: n = 96; 44.9%	*Intervention profile*: multidisciplinary treatment *Intervention time*: 3 w, generally 4–5 therapy sessions a day on workdays *Details*: combination of physical, psychological, educational and work-related treatment sessions *Setting*: inpatient
Heiskanen et al. (2012)	Finland	Cohort study	D: chronic non-malignant pain n: r, nr 99, 96 age r/nr 52.5 (14.6), 56.4 (15.6) female: r, nr 64%, 52% duration r, nr: <1 year: 8%, 6% 1–5 years: 47%, 46% ≥6 years: 44%, 48%	*Intervention profile*: multidisciplinary pain treatment *Intervention time*: not reported *Details*: pain management was individually designed and consisted of diagnostic evaluation and at least two of the following: analgesic medication, local analgesia, spinal cord stimulation, physiotherapeutic counselling and exercise programs, psychological evaluation, supportive psychological therapy, teaching of pain management strategies and socioeconomic counselling *Setting*: inpatient
Martin et al. (2014)	Spain	RCT	D: fibromyalgia n: 54 age: 48.68 (8.68) female: 90.74% duration: 177.4 (119.6) mo	*Intervention profile*: interdisciplinary treatment *Intervention time*: 12 sessions in 6 w *Details*: psychological component: focused on CBT interventions; educational component: addressed topics related to the characteristics of FM such as the nature of the condition, its usual course, treatment possibilities, appropriate organization of daily activities and the physician-patient relationship; physiotherapeutic component: progressive physical training with warming, stretching and muscle strengthening exercises and without machine weights *Setting*: outpatient
Martin et al. (2017)	Spain	Cohort study	D: fibromyalgia n: 138 age: 50.09 (9.27) female: 92.75% duration: 168.1 (119.6) mo	*Intervention profile*: interdisciplinary treatment *Intervention time*: 12 sessions in 6 w *Details*: educational activities focused on better understanding FM; CBT to target the cognitive, physiological, and behavioral domains of FM; a physiotherapeutic component that included appropriate warm-up, exercise and stretching routines *Setting*: outpatient
Moradi et al. (2010)	Germany	Cohort study	D: chronic musculoskeletal pain n: 389 age: 44.3 (9.1) female: 57% duration: 26.7 (25.9) mo	*Intervention profile*: multidisciplinary therapy *Intervention time*: 3 w; 6 h session on 5 days each w; total of 90 h *Details*: physical exercises, ergonomic training, psychotherapy, patient education, behavioral therapy and workplace-based interventions on an individual basis and in group sessions *Setting*: inpatient
Orenius et al. (2013)	Finland	Cohort study	D: chronic musculoskeletal pain n: 111 age: 45 (8) female: 65% duration: ≤1 year: 5; 5% 1–5 years: 76; 68% ≥5 years: 30; 27%	*Intervention profile*: multidisciplinary pain management program *Intervention time*: 19 days, in three phases (3 + 13 + 3) during 6–7 mo *Details*: physical and functional exercises (water gymnastics, gym exercises, relaxation and flexibility training), evaluation of the social situation, psychological assessment of pain-related stress factors and personal pain management training, including mindfulness and relaxation practicing *Setting*: outpatient
Tseli et al. (2020)	Sweden	Cohort register based (SQRP)	D: chronic pain n: 2876 age: 43.5 (10.7) female: 76.8% duration: 106.2 (107.7) mo	*Intervention profile*: interdisciplinary multimodal pain rehabilitation *Intervention time*: on average 10 w *Details*: pain education, supervised physical activity, training in simulated environments, CBT *Setting*: outpatient

age = age (mean); CBT = Cognitive Behavioral Therapy; d/w = days per week; duration = months (mean); D = Diagnosis; FM = Fibromyalgia; n = number of participants; ns = non-responders; mo = month(s); r = responders; RCT = Randomized Controlled clinical Trial; SQRP = Swedish Quality Register for Pain Rehabilitation; w = week(s).

The intervention profiles of the included studies were described as multidisciplinary (pain) treatment [33–35, 37], interdisciplinary treatment/therapy [38, 39, 41], interdisciplinary multimodal pain rehabilitation [42, 44], multidisciplinary rehabilitation/pain management program [32, 40], multimodal therapy/rehabilitation program [36, 43] and inpatient interdisciplinary program [31]. Table 1 describes the intervention details of the included studies.

### Outcome Measures

Generic or disease-specific measures for the outcome hrQoL were extracted. Questionnaires used in the included studies were the Fibromyalgia Impact Questionnaire (FIQ) [38, 41], the 15-dimensional health-related quality of life measure (15D) [37, 40], the 36-Item Short Form Health Survey (SF-36) with the subscales Mental Health (MH) and Physical Health (PF) [31, 32, 36, 39], the SF-36 subscales Physical Component Summary (PCS) and Mental Component Summary (MCS) [33–35, 42]. One study [43] combined the outcome PCS with a subscale of the Multidimensional Pain Inventory (MPI) and one study [44] combined the Mental Component Summary with the Hospital Anxiety and Depression Scale (HAD). The outcome of interest, hrQoL was grouped into four categories: physical hrQoL, mental hrQoL, FIQ, and 15D.

### Prognostic Factors

In total, 49 different prognostic factors were identified. Pain-related factors were mostly assessed with, for example, the Numeric scale 7 days (NRS-7d), pain duration in years or the pain region index (PRI). Examples of measurement instruments used for psychological-related factors include the Hospital Anxiety and Depression Scale (HADS) for emotional distress. For cognitive behavioral negative factors, for example, the Tampa Scale of Kinesiophobia (TSK), and the Pain Catastrophizing Scale (PCS) was used and correspondingly, for cognitive behavioral positive factors the Coping Strategies Questionnaire (CSQ) and the Pain Self-Efficacy Questionnaire (PSEQ). All prognostic physical functioning-related factors were self-reported such as the SF-36 PF.

### Risk of Bias Within Studies

Nine out of the 14 studies were classified as studies with a high RoB, while one study was classified as having moderate RoB [34] and four studies [31, 32, 35, 36] were rated as having low RoB (Table 2). Seven studies were classified as having high RoB in the domain Attrition, one additional study in the domain of Study Confounding, resulting in total nine studies with high RoB [33, 37–44].

**Table 2.** Risk of Bias within studies

References	Study Participation	Study Attrition	Prognostic Factor Measurement	Outcome Measurement	Study Confounding	Statistical Analysis and Reporting	Overall Risk of Bias
Angst et al. (2014)	low	moderate	low	low	low	low	low
Bremander et al. (2011)	low	moderate	low	low	low	low	low
Buchner et al. (2007)	moderate	low	low	low	high	low	high
Dong et al. (2019)	moderate	high	low	low	low	low	high
Farin et al. (2013)	moderate	moderate	low	low	low	low	moderate
Gerdle et al. (2016)	moderate	high	low	low	low	low	high
Glattacker et al. (2010)	low	moderate	low	low	low	low	low
Glattacker et al. (2018)	low	moderate	low	low	low	low	low
Heiskanen et al. (2012)	moderate	high	low	low	high	moderate	high
Martin et al. (2014)	low	high	low	low	low	low	high
Martin et al. (2017)	low	high	low	low	low	low	high
Moradi et al. (2010)	high	high	moderate	low	moderate	low	high
Orenius et al. (2013)	moderate	moderate	low	low	low	moderate	high
Tseli et al. (2020)	moderate	high	low	low	low	low	high

### Synthesis of the Results

The detailed results (statistical analyses and included covariates in the individual multivariate models) from the included studies are presented in Supplementary Data (Table S2). Given the heterogeneity in the measurement constructs and statistical models, a meta-analysis would not have provided meaningful interpretable information and therefore, only a narrative approach to data synthesis was conducted [13]. The results were synthesized separately for the outcomes physical hrQoL, mental hrQoL, FIQ, and 15D, and grouped by prognostic factor.

The modified GRADE assessment is presented in Table 8. All included studies are primary (phase one) studies and therefore the certainty of evidence was downgraded for “phase of investigation”. We also downgraded all prognostic factors on “publication bias” because none of the prognostic factors has been investigated in a larger number of cohort studies and there is no evidence that the prognosis research is not affected by publication bias [30]. None of the prognostic factors could be upgraded for the domain “moderate or large effect” and “exposure-gradient response”.

### Pain-Related Factors

The narrative analyses concerning the association between *pain intensity* and the outcome physical hrQoL was assessed in seven studies [31, 32, 34, 35, 42–44] and showed inconsistent results (Table 3). One study [31] showed that high pain levels at baseline was statistically significantly associated with positive effects on hrQoL, while three studies [34, 42, 44] showed the opposite. Three studies reported no significant association. Out of the statistically non-significant associations, two showed opposite directions [32, 35], and one displayed no results [43]. Concerning the association between pain intensity and the outcome mental hrQoL, five studies [32, 34, 35, 42, 44] showed inconsistent results. There were two studies [42, 44] with results in a negative and two studies [32, 35] in a positive direction, but only one [42] of the five studies showed a statistically significant negative association, indicating that lower pain levels at baseline predicted a better outcome in mental hrQoL. The study investigating for the outcome 15D [37] found no statistically significant association, but a positive direction. The results remained inconsistent, even when subgrouping for the different overall RoB, the different follow-up times, or the different analyses or the sample size.

**Table 3:** Narrative analyses of pain-related factors

Authors	Outcome	Follow-up	Instrument	Association	Direction	Analysis	Effect Size	*P* value
			**Pain intensity**					
Angst et al. 2014	p hrQoL	6 mo	SF-36 bodily pain	+	+	multivariate regression	β: 0.202	.023
Bremander et al. 2011		6 mo	VAS 0–100	0	−	multivariate regression	OR (95% CI): 0.9 (0.4–2.0)	.72
Dong et al. 2019		12 mo	NRS-7d	−	−	multivariate regression	β (SE): −3.29 (0.15)	<.01
Farin et al. 2013		6 mo	VAS 0–100	−	−	multivariate regression	β: −0.042	<.001
Gerdle et al. 2016		12 mo	NRS-7d	0	NA	multivariate regression	NA	ns
			MPI pain severity	0	NA		NA	ns
Glattacker et al. 2018		6 mo	VAS 0–100	0	+	multivariate regression	β: 0.052	.264
Tseli et al. 2020		12 mo	NRS-7d	−	−	multivariate regression	OR (95% CI): 0.92 (0.87–0.97)	.001
Bremander et al. 2011	m hrQoL	6 mo	VAS 0–100	0	+	multivariate regression	OR (95% CI): 1.5 (0.1–1.5)	.18
Dong et al. 2019		12 mo	NRS-7d	−	−	multivariate regression	β (SE): −1.31 (0.17)	<.01
Farin et al. 2013		6 mo	VAS 0–100	0	NA	multivariate regression	NA	ns
Glattacker et al. 2018		6 mo	VAS 0–100	0	+	multivariate regression	B: 0.092	.057
Tseli et al. 2020		12 mo	NRS-7d	0	−	multivariate regression	OR (95% CI): 0.96 (0.91–1.01	.106
Heiskanen et al. 2012	15D	12 mo	VAS 0–100	0	+	ANOVA	mean (SD) r: 64 (25),	ns
							mean (SD) nr: 62 (26)	
			**Pain duration**					
Buchner et al. 2007	p hrQoL	6 mo	Grade of chronicity	−	−	ANOVA	mean (SD): 82.02(47)	.03
			Stage I and II				mean (SD): 72.58 (22.8)	
			Stage III				mean (SD): 75.00 (25.2)	
			Stage IV					
Farin et al. 2013		6 mo	Duration <2 y	0	NA	multivariate regression	NA	ns
Glattacker et al. 2010		6 mo	Illness <1 year	0	−	multivariate regression	β: −0.046	ns
			1–2 years				β: −0.050	ns
			3–5 years				β: −0.014	ns
			6–10 years				β: −0.023	ns
Tseli et al. 2020		12 mo	months	0	NA	multivariate regression	NA	ns
Buchner et al. 2007	m hrQoL	6 mo	Grade of chronicity	0	NA	ANOVA	mean (SD): 60.58 (8.1)	.494
			Stage I and II				mean (SD): 61.96 (7.4)	
			Stage III				mean (SD): 61.40 (7.90)	
			Stage IV					
Farin et al. 2013		6 mo	Duration <2 y	0	NA	multivariate regression	NA	ns
Glattacker et al. 2010		6 mo	Illness <1 year	0	−	multivariate regression	β: −0.135	ns
			1–2 years				β: −0.158	ns
			3–5 years				β: −0.058	ns
			6–10 years				β: −0.023	ns
Tseli et al. 2020		12 mo	months	0	NA	multivariate regression	NA	ns
Martin et al. 2014	FIQ	6 mo	n of years	0	NA	univariate analysis	NA	ns
Martin et al. 2017		12 mo	n of years 6–10	+	+	multivariate regression	β (SE): 0.20 (0.07)	.003
			11–15				β (SE): 0.19 (0.03)	.03
			>15				β (SE): 0.22 (0.06)	.0003
Heiskanen et al. 2012	15D	12 mo	<1 years	0	NA	Pearson’s χ^2^ test	r: 8%, nr: 6%	ns
			1–5 years				r: 47%, nr: 46%	
			≥6 years				r: 44%, nr: 48%	
			**Pain sites**					
Bremander et al. 2011	p hrQoL	6 mo	Pain mannequin	0	+	multivariate regression	OR (95% CI): 1 (0.2–2.2)	.6
			7–13					.47
			13–18				OR (95% CI): 2 (0.5–4.9)	
Dong et al. 2019		12 mo	PRI	−	−	multivariate regression	β (SE): −0.33 (0.05)	<.05
Moradi et al. 2010		6 mo	n of pain sites	−	−	ANOVA	mean (SD): 78.4 (18.1)	1/2: ns
			1: single-site				mean (SD): 79.2 (19.7)	1/3: .006
			2: dual-sites				mean (SD): 70.1 (24.5)	2/3: .044
			3: multiple-sites					
Tseli et al. 2020		12 mo	n of pain regions	0	−	multivariate regression	OR (95% CI): 0.97 (0.61–1.53)	.889
			3–6				OR (95% CI): 0.67 (0.43–1.06)	.087
			>7, not CWP				OR (95% CI): 0.69 (0.45–1.06)	.088
			CWP					
Bremander et al. 2011	m hrQoL	6 mo	Pain mannequin	0	−	multivariate regression	OR (95% CI): 0.6 (0.2–1.8)	.34
			7–13		+		OR (95% CI): 1.7 (0.6–5.4)	.34
			13–18					
Dong et al. 2019		12 mo	PRI	−	−	multivariate regression	β (SE): −0.17 (0.05)	<.01
Moradi et al. 2010		6 mo	n of pain sites	0	−	ANOVA	mean (SD): 61 (7.2)	1/2: ns
			1: single-site				mean (SD): 62.1 (7.4)	1/3: ns
			2: dual-sites				mean (SD): 60.1 (8.2)	2/3: ns
			3: multiple-sites					
Tseli et al. 2020		12 mo	n of pain regions	0	NA	multivariate regression	NA	ns

ANOVA = ANalysis Of VAriance; Association = significant association; B = regression coefficient; β = standardized regression coefficient; direction = not significant association; CWP = Chronic Widespread Pain; FIQ = Fibromyalgia Impact Questionnaire; m hrQol = mental health-related quality of life; mo = months; MPI = Multidimensional Pain Inventory; n = number; NA = not available; nr = non-responders; NRS 7d = Numeric Rating Scale 7 days; ns = not significant; OR (95% CI) = Odds Ratio (95% Confidence Interval); p hrQoL = physical health-related quality of life; PRI = Pain Region Index r = responders; SD = Standard Deviation; SE = Standard Error; SF-36 = 36-Item Short Form Health Survey; VAS = Visual Analog Scale; 15D = 15-dimensional health-related quality of life.

In summary, the results on the association between pain intensity at baseline and hrQoL at follow-up was found to be inconsistent, since both higher and lower levels of pain intensity at baseline were associated with positive outcomes. The GRADE analyses showed that the certainty of evidence for this finding was very low (Table 8).

*Pain duration* at baseline displayed almost no associations with the outcomes physical and mental hrQoL in four studies [33, 34, 36, 44] (Table 3). Two studies [33, 36] pointed in the direction that a lower stage of chronicity was associated with a better outcome in physical hrQoL, but only one [33] showed a statistically significant association. For the outcome mental hrQoL, one study [36] reported a negative direction, but there were no statistically significant associations [33, 34, 36, 44]. For the FIQ outcome, one study [38] showed that a longer pain duration at baseline was statistically significantly associated with a better outcome, while another study [41] showed no association. There was no predictive value for pain duration at baseline for the outcome 15D [37]. Sensitivity analyses did not alter the results that pain duration had almost no predictive value for hrQoL more than 6 months after IPR and the GRADE analyses resulted in very low certainty of evidence due to additional downgrading on “inconsistency” (Table 8).

The analyses of the four studies [32, 39, 42, 44] on the number of *pain sites* indicated that patients with fewer pain sites at baseline had a better outcome for hrQoL (Table 3). For physical hrQoL three studies [39, 42, 44] showed a direction favors fewer pain sites, and two studies out of these displayed statistically significant associations. One study [42] showed a statistically significant association favoring less pain sites at baseline for the outcome mental hrQoL. Three studies [32, 39, 44] showed no statistically significant associations but a negative direction [39], and one [32] study showed both negative and positive directions depending on the number of pain sites. The negative associations were not reinforced in the subgroup analyses mentioned earlier. The GRADE analyses resulted in low certainty of evidence that fewer pain sites at baseline could predict a better outcome for hrQoL at follow-up (Table 8).

### Psychological Related Factors

The narrative analyses regarding the psychological predictors are shown in Tables 4–6. Inconsistent results were found for the predictive value of measurements for *emotional distress* (Table 4). Six studies [31, 32, 35, 42–44] investigated the outcome physical hrQoL and three studies [31, 32, 44] found statistically significant positive associations between higher emotional distress at baseline and increased physical hrQoL [31, 32, 44]. One study [42] showed a statistical negative association and five studies [31, 35, 42–44] did not report any associations. For the outcome mental hrQoL, two studies [32, 36] reported that higher emotional distress at baseline was associated with increased mental hrQoL while three studies [35, 42, 44] showed the opposite. The study using the FIQ as outcome measure [38], showed no association or a statistically significant positive association between emotional distress at baseline and increased hrQoL, similar to the study using outcome 15D [40]. The results for emotional distress as a prognostic factor remained inconsistent even in the subgroup analyses. Moreover, even the different constructs of measurement as HADS-A or HADS-D showed conflicting results. Based on the GRADE analyses, the certainty of evidence for the inconsistent results for the association between emotional distress and the outcome hrQoL after IPR is very low (Table 8).

**Table 4.** Narrative analyses of psychological factors—emotional distress

Authors	Outcome	Follow-up	Instrument	Association	Direction	Analysis	Effect size	*P* value
Angst et al. 2014	p hrQoL	6 mo	HADS-D	+	+	multivariate regression	β: 0.25	<.001
			HADS-A	0	NA		NA	ns
Bremander et al. 2011		6 mo	HADS-A	+	+	multivariate regression	OR (95% CI): 2.6 (1.0–6.8)	.05
			HADS-D	+	+		OR (95% CI): 5.6 (1.5–21.7)	.01
Dong et al. 2019		12 mo	HADS-A	0	NA	multivariate regression	NA	ns
			HADS-D	−	−		β (SE): −1.20 (0.09)	<.01
Gerdle et al. 2016		12 mo	HADS-A	0	NA	multivariate regression	NA	ns
			HADS-D	0	NA		NA	ns
			MPI distress	0	NA		NA	ns
Glattacker et al. 2018		6 mo	HADS-A	0	NA	multivariate regression	NA	ns
			HADS-D	0	NA		NA	ns
Tseli et al. 2020		12 mo	SF-36 MCS	+	+	multivariate regression	OR (95% CI): 1.02 (1.01–1.03)	0.003
			HADS-A	+	+		OR (95% CI): 1.03 (1.01–1.05)	.019
			HADS-D	0	NA		NA	ns
Bremander et al. 2011	m hrQoL	6 mo	HADS-A	0	+	multivariate regression	OR (95% CI): 1.4 (0.6–3.4)	.45
			HADS-D	+	+		OR (95% CI): 3.6 (1.2–10.2)	.02
Dong et al. 2019		12 mo	HADS-A	−	−	multivariate regression	β (SE): −1.44 (0.11)	<.01
			HADS-D	−	−		β (SE): −2.52 (0.10)	<.01
Glattacker et al. 2010		6 mo	SF-36 MH	+	+	multivariate regression	β: 0.611	<.001
Glattacker et al. 2018		6 mo	SF-12 MCS	0	−	multivariate regression	B: −0.074	.05
			HADS-A	0	−		B: −0.284	.385
			HADS-D	−	−		B: −0.989	.007
Tseli et al. 2020		12 mo	SF-36 MCS	−	−	multivariate regression	OR (95% CI): 0.92 (0.92–0.93)	.000
			HADS-A	0	NA		NA	ns
			HADS-D	0	NA		NA	ns
Martin et al. 2017	FIQ	12 mo	HADS-A 8–10	+	+	multivariate regression	β (SE): 0.30 (0.13)	.02
			≥ 11				β (SE): 0.23 (0.12)	.04
			HADS-D 8–10	0	−	univariate analysis	β (SE): −0.01 (0.07)	.5
			≥ 11				β (SE): −0.05 (0.07)	.87
Orenius et al. 2013	15D	12 mo	BAI	−	−	multivariate regression	OR (95% CI): 0.19 (0.07–0.53)	NA
			BDI	+	+		OR (95% CI): 2.72 (0.97–7.66)	NA

Association = significant association; B = regression coefficient; β = standardized regression coefficient; BAI/BDI = Beck Anxiety and Depression Inventory; direction = not significant association; FIQ = Fibromyalgia Impact Questionnaire; HADS = Hospital Anxiety and Depression Scale; MCS = Mental Component Summary; MH = Mental Health; m hrQol = mental health-related quality of life; mo = months; MPI = Multidimensional Pain Inventory; NA = not available; ns = not significant; OR (95% CI) = Odds Ratio (95% Confidence Interval); p hrQoL = physical health-related quality of life; SE = standard error; SF-36/12 = 36/12-Item Short Form Health Survey; 15D = 15-dimensional health-related quality of life.

**Table 5.** Narrative analyses of psychological factors—cognitive behavioral negative

Authors	Outcome	Follow-up	Instrument	Association	Direction	Analysis	Effect size	*P* value
Angst et al. 2014	p hrQoL	6 mo	CSQ	0	NA	multivariate regression	NA	ns
Farin et al. 2013		6 mo	FABQ-workb	−	−	multivariate regression	β: −0.731	<.001
			LOC-FE	0	NA		NA	ns
Gerdle et al. 2016		12 mo	TSK	0	NA	multivariate regression	NA	ns
Glattacker et al. 2010		6 mo	IPQR: TL	0	0	multivariate regression	β: 0	ns
			IPQR: ID	0	−		β: −0.088	ns
			IPQR: CO	0	−		β: −0.114	ns
			IPQR: ER	0	+		β: 0.07	ns
Glattacker et al. 2018		6 mo	IPQR: TL	0	−	multivariate regression	B: −0.286	.15
			IPQR: ID	0	NA		NA	ns
			IPQR: CO	0	NA		NA	ns
			IPQR: ER	0	NA		NA	ns
			FABQ-cause	0	NA		NA	ns
			FABQ-prog	0	NA		NA	ns
			FABQ-PA	0	NA		NA	ns
			PCS	0	NA		NA	ns
Farin et al. 2013	m hrQoL	6 mo	FABQ-workb	−	−	multivariate regression	β: −0.556	<.001
			LOC-FE	−	−		β: −0.098	.013
Glattacker et al. 2010		6 mo	IPQR: TL	0	+	multivariate regression	β: 0.128	ns
			IPQR: ID	0	−		β: −0.122	ns
			IPQR: CO	0	−		β: −0.006	ns
			IPQR: ER	0	−		β: −0.055	ns
Glattacker et al. 2018		6 mo	IPQR: TL	0	NA	multivariate regression	NA	ns
			IPQR: ID	0	NA		NA	ns
			IPQR: CO	0	−		B: −0.225	.45
			IPQR: ER	0	−		B: −0.018	.943
			FABQ-cause	0	−		B: −0.841	.224
			FABQ-prog	0	−		B: −0.359	.626
			FABQ-PA	0	NA		NA	ns
			PCS	0	−		B: −0.213	.185
Orenius et al. 2013	15D	12 mo	TSK	0	NA	multivariate regression	NA	ns

Association = significant association; B = regression coefficient; β = standardized regression coefficient; CSQ = Coping Strategies Questionnaire; direction = not significant association; FABQ = Fear Avoidance Belief Questionnarie; IPQR = Illness Perception Questionnaire; LOC = Control Beliefs Concerning Illness and Health Questionnaire; m hrQol = mental health-related quality of life; mo = months; NA = not available; ns = not significant; PCS = Pain Catastrophizing Scale; p hrQoL = physical health-related quality of life; TSK = Tampa Scale of Kinesiophobia; 15D = 15-dimensional health-related quality of life.

**Table 6.** Narrative analyses of psychological factors—cognitive behavioral positive

Authors	Outcome	Follow-up	Instrument	Association	Direction	Analysis	Effect Size	*P* value
Farin et al. 2013	p hrQoL	6 mo	LOC-I	0	NA	multivariate regression	NA	ns
			IPQR: C	0	NA		NA	ns
Gerdle et al. 2016		12 mo	CPAQ-AE	0	NA	multivariate regression	NA	ns
			CPAQ-PW	0	NA		NA	ns
			MPI LifeCon	0	NA		NA	ns
			RTW-expect	+	+		VIP: 1.48	sig
			RTW- prognosis	+	+		VIP: 1.38	sig
Glattacker et al. 2010		6 mo	IPQR: PC	0	+	multivariate regression	β: 0.018	ns
			IPQR: TC	0	+		β: 0.063	ns
			IPQR: C	0	+		β: 0.015	ns
			GSES	0	−		β: −0.069	ns
Glattacker et al. 2018		6 mo	IPQR: PC	0	−	multivariate regression	B: 0.268	.283
			IPQR: TC	0	NA		NA	ns
			IPQR: C	0	NA		NA	ns
			CPQ	0	NA		NA	ns
			PSEQ	+	+		B: 0.209	.018
Tseli et al. 2020		12 mo	Bel. of rh 2	+	+	multivariate regression	OR (95% CI): 1.09 (0.87–1.37)	.445
			3				OR (95% CI): 1.29 (1.03–1.60)	.024
			4				OR (95% CI): 1.85 (1.43–2.40)	.000
			5				OR (95% CI): 2.43 (1.77–3.33)	.000
			MPI: LC	0	NA		NA	ns
Farin et al. 2013	m hrQoL	6 mo	LOC-I	+	+	multivariate regression	β: 0.077	.049
			IPQR: C	+	+		β: 0.118	.05
Glattacker et al. 2010		6 mo	IPQR: PC	0	+	multivariate regression	β: 0.014	ns
			IPQR: TC	0	+		β: 0.083	ns
			IPQR: C	0	−		β: −0.068	ns
			GSES	0	+		β: 0.02	ns
Glattacker et al. 2018		6 mo	IPQR: PC	0	NA	multivariate regression	NA	ns
			IPQR: TC	0	NA		NA	ns
			IPQR: C	0	NA		NA	ns
			CPQ	0	NA		NA	ns
			PSEQ	0	+		B: 0.011	.916
Tseli et al. 2020		12 mo	Bel. of rh	0	NA	multivariate regression	NA	ns
			MPI: LC	+	+		OR (95% CI): 1.23 (1.12–1.35)	.000
Martin et al. 2014	FIQ	6 mo	CAD-R	0	+	multivariate regression	β (SE): 0.87 (0.46)	.07

Association = significant association; B = regression coefficient; β =standardized regression coefficient; Bel. of rh = Belief of restored health question; direction = not significant association; CAD-R = Coping with Chronic Pain Questionnaire; CPAQ = Chronic Pain Acceptance Questionnaire; CPQ = Coping Procedure Questionnaire; FIQ = Fibromyalgia Impact Questionnaire; GSES = General Self-efficacy scale; IPQR = Illness Perception Questionnaire; LOC = Control Beliefs Concerning Illness and Health Questionnaire; m hrQol = mental health-related quality of life; mo = months; MPI = Multidimensional Pain Inventory; NA = not available; ns = not significant; OR (95% CI) = Odds Ratio (95% Confidence Interval); p hrQoL = physical health-related quality of life; PSEQ = Pain Self-Efficacy Questionnaire; RTW-expect = expectation of Return To Work = RTW-prognosis = perceptions of prognosis on Return To Work; SE = Standard Error; sig = significant; VIP = Variable Influence on Projection.

In six studies [31, 34–36, 40, 43] *cognitive behavioral negative* factors showed lower levels of cognitive behavioral negative factors at baseline predicted a better outcome for hrQoL (Table 5). One [34] of the five studies [31, 34–36, 43] showed a statistically significant association between lower levels of cognitive behavioral negative factor and the outcome physical hrQoL. The other studies showed no statistically significant associations, but the studies that reported directions were all in favor of low levels of cognitive behavioral negative factors except one. For the outcome mental hrQoL, one study [34] showed significant negative associations and two studies [35, 36] showed no association, but of the reported results all except one showed negative directions. There was no association for the outcome 15D [40] and cognitive behavioral negative factors at baseline. The negative associations for mental and physical hrQoL were from the same study with the measurement FABQ-work and LOC-FE. A large proportion of the variables consisted of different scales of one measurement instrument (IPQR), which did not show a significant association in any subscale. The GRADE analyses showed low certainty of evidence, that lower levels of cognitive behavioral negative factors at baseline predict a better outcome for hrQoL (Table 8).

*Cognitive behavioral positive* prognostic factors in six studies [34–36, 43, 44] showed, that a better outcome of hrQoL was seen in those patients with higher level of cognitive behavioral positive factors at baseline. In three studies [35, 43, 44] statistically significant increased physical hrQoL at follow-up was found in patient with a higher level of cognitive behavioral positive factors at baseline. Two studies [34, 44] showed that higher levels of positive cognitive behavioral factors were associated with a better outcome regarding mental hrQoL. These findings for physical and mental health were supported by two studies [35, 36] who showed associations in the positive direction, although not statistically significant. Martin et al. showed no significant result for the outcome FIQ but as well a positive direction [41]. Subgroup analyses did not affect the results. The GRADE analyses found low certainty of evidence for the association between higher levels of cognitive behavioral positive factors at baseline and increased hrQoL after IPR.

### Physical Functioning-Related Factors

Five studies [31, 35, 36, 43, 44] investigated the association between *self-reported physical functioning* at baseline and the outcome hrQoL, with inconsistent results (Table 7). Lower self-reported physical functioning at baseline predicted a significant better outcome in physical hrQoL in three studies [31, 43, 44], but in two other studies [35, 36] the opposite was found, while one study [43] showed no association. For the outcome mental hrQoL, none of the two outcome measurements that were used displayed any associations with initial self-reported physical functioning. The results remained inconsistent when analyzing individual measurements for example SF-PF or MPI-PI separately. Due to additional downgrading on “inconsistency” the GRADE analyses showed a very low certainty of evidence for the findings that lower physical functioning at baseline is inconsistent in predicting hrQoL at follow-up (Table 8).

**Table 7.** Narrative analyses physical functioning-related factors

Authors	Outcome	Follow-up	Instrument	Association	Direction	Analysis	Effect size	*P* value
			**Self-reported function**					
Angst et al. 2014	p hrQoL	6 mo	SF-36 PF	−	−	multivariate regression	β: −0.622	<.001
Gerdle et al. 2016		12 mo	MPI-PI	0	NA	multivariate regression	NA	ns
			SF-36 PCS	−	**−**		VIP: 1.07	sig
Glattacker et al. 2010		6 mo	SF-36 PF	+	+	multivariate regression	β: 0.621	<.001
Glattacker et al. 2018		6 mo	SF-12 PCS	+	+	multivariate regression	B: 0.468	<.001
Tseli et al. 2020		12 mo	SF-36 PCS	−	−	multivariate regression	OR (95% CI): 0.92 (0.91–0.94)	.000
			MPI-PI	−	**−**		OR (95% CI): 0.85 (0.76–0.95)	.004
Tseli et al. 2020	m hrQoL	12 mo	MPI-PI	0	NA	multivariate regression	NA	ns
			SF-36 PCS	0	NA		NA	ns

Association = significant association; B = regression coefficient; β = standardized regression coefficient; direction = not significant association; m hrQol = mental health-related quality of life; mo = months; MPI-PI = Multidimensional Pain Inventory-pain interference; NA = not available; ns = not significant; OR (95%-CI) = Odds Ratio (95%-Confidence Interval); PCS = Physical Component Summary; PF = Physical Health; p hrQoL = physical health-related quality of life; SF-36/12 = 36/12-Item Short Form Health Survey; sig = significant; VIP = Variable Influence on Projection.

**Table 8.** Overall quality of evidence assessed with the adapted GRADE approach

Potential Prognostic Factors Identified	Number of Participants	Number of Studies	Phase of Investigation	Study Limitations	Inconsistency	Indirectness	Imprecision	Publication Bias	Moderate/large Effect Size	Dose Effect	Overall Quality
Outcome physical hrQoL											
pain intensity	5,176	7	1	√	×	√	√	×	×	×	+
pain duration	4,196	4	1	√	×	√	√	×	×	×	+
pain sites	4,234	4	1	×	√	√	√	×	×	×	++
emotional distress	4,461	6	1	√	×	√	√	×	×	×	+
behavioral negative	1,549	5	1	√	√	√	√	×	×	×	++
behavioral positive	4,250	5	1	√	√	√	√	×	×	×	++
physical functioning	3,737	5	1	√	×	√	√	×	×	×	+
Outcome mental hrQoL											
pain intensity	4,747	5	1	√	×	√	√	×	×	×	+
pain duration	4,196	4	1	√	×	√	√	×	×	×	+
pain sites	4,234	4	1	×	√	√	√	×	×	×	++
emotional distress	4,304	5	1	√	×	√	√	×	×	×	+
behavioral negative	1,147	3	1	√	√	√	√	×	×	×	++
behavioral positive	4,023	4	1	√	√	√	√	×	×	×	++
physical functioning	2,876	1	1	√	×	√	√	×	×	×	+
Outcome FIQ											
pain duration	192	2	1	√	×	√	√	×	×	×	+
emotional distress	138	1	1	√	×	√	√	×	×	×	+
behavioral positive	54	1	1	√	×	√	×	×	×	×	+
Outcome 15D											
pain intensity	195	1	1	×	×	√	√	×	×	×	+
pain duration	195	1	1	×	×	√	×	×	×	×	+
emotional distress	111	1	1	×	×	√	×	×	×	×	+
behavioral negative	111	1	1	×	×	√	√	×	×	×	+

behavioral negative = cognitive behavioral negative factors, behavioral positive = cognitive behavioral positive factors; FIQ = Fibromyalgia Impact Questionnaire; GRADE = Grading of Recommendations Assessment, Development and Evaluation; hrQoL = health-related quality of life; × = serious limitations; √ = no serious limitations; + = very low level of evidence; ++ = low level of evidence.

## Discussion

This review showed that pain intensity, emotional distress and physical functioning at baseline is inconsistent for prediction of hrQoL, 6 or more months after IPR in chronic pain patients. Additionally, pain duration has no predictive value for this outcome. Furthermore, we found that fewer pain sites can predict a better outcome, that lower levels of cognitive behavioral negative factors, and that higher levels of cognitive behavioral positive factors predict a better outcome of hrQoL. However, the certainty of evidence was low to very low, therefore no firm conclusion on prognostic ability of these factors can be drawn.

### Pain-Related Factors

The results of previous reviews regarding pain intensity as a predictor are also inconclusive: van der Hulst et al. showed that high pain intensity at baseline had a negative predictive value in chronic low back pain patients for the outcome activity and participation limitations [19], de Rooij et al. revealed the opposite in fibromyalgia patients [17], and Artus et al. showed that high pain intensity is considered to be a generic prognostic factor for poor prognosis in low back pain patients in primary care [14].

Regarding pain duration our review showed that there is very low certainty of evidence that pain duration did not have predictive value, and similar to the findings of van der Hulst et al. in fibromyalgia patients for the outcome activity limitations and participation restrictions [19]. Neither did pain duration predict physical functioning in Tseli et al. [18]. However, when looking at the direction of our results alone, we saw the same tendency as Mills et al. who found that having longer pain duration at baseline predicted poorer hrQoL [45]. We found very low certainty of evidence that less pain sites at baseline predict a better outcome for hrQoL 6 month after IPR. These results are supported by findings that widespread pain is a generic prognostic factor for poor prognosis in primary care low back pain patients [14]. Furthermore, widespread pain has been shown to be associated with a longer duration of pain and a more severe clinical picture at baseline [46]. Our results on the predictive value of these different pain related aspects however emerged inconsistent, which may partly have occurred through our chosen methodological approach.

In summary, caregivers should be aware that pain intensity levels may not influence the prognosis. These results affirm the biopsychosocial approach of IPR, which primarily aims to restore physical and psychological functioning, whereas pain reduction is a secondary objective [47]. However, our results suggest that regarding hrQoL, it is more difficult to benefit from IPR when multiple sites of pain are present.

### Psychological Related Factors

In our review, we found with a very low certainty of evidence that emotional distress, such as depression, at baseline had inconsistent predictive value for hrQoL at follow-up. These results are in line with the findings of van der Hulst et al. [19]. However, another review showed moderate evidence for low baseline emotional distress and a positive outcome regarding physical functioning [18]. Moreover, de Rooij et al. found that a major depression predicted a poorer outcome in hrQoL in fibromyalgia patients with the reasonable conclusion that patients with emotional problems respond less well to interdisciplinary treatment [17]. This discrepancy between our study and the other reviews could be explained by our large heterogeneity and methodological differences between included studies.

We found low certainty of evidence favoring low levels of negative behavioral factors, such as fear avoidance and pain catastrophizing, as predictors for increased hrQoL outcomes. These factors were also shown of importance for other outcomes of previous reviews [18], such as physical functioning and chronic pain [45]. Moreover, they were of importance in a review investigating predictors in the transition from acute to chronic pain [48].

In our review, high initial cognitive behavioral positive factors such as self-efficacy showed a better outcome for hrQoL at follow-up. These results are supported by previous reviews on mixed chronic pain population [49, 50]. Somers et al. stated that patients with low baseline self-efficacy may not be able to implement the strategies and behavioral changes necessary to achieve improved hrQoL [51]. The results of our review support the current knowledge that patients with high baseline levels of these positive factors could use these as a resource in therapy and benefit more from IPR in terms of hrQoL at follow-up.

Our results support the current evidence that psychological factors are strongly related to chronic pain [52]. Chronic pain patients have alterations in brain regions involved with cognitive and emotional processes [53, 54] and imaging studies have confirmed that attention state, positive and negative emotions and other factors unrelated to the pain stimulus itself, alter the activity of afferent and descending pain pathways [55]. Based on our results, we confirm the current evidence to target these processes already in early treatment and continuously throughout the entire rehabilitation process [50, 56].

### Physical Function-Related Factors

In our review, results for baseline self-reported physical function as predictors for hrQoL are inconsistent. However, previous systematic reviews conclude that exercise and physical activity as intervention have positive effects in terms of improved quality of life in chronic pain patients [57, 58]. However, our inconsistent results are based on low certainty of quality, suggesting that further studies may influence these results. Moreover, no studies had investigated performance-based physical function as a prognostic factor for hrQoL. Thus, further studies are needed to explore the predictive value of preferably objectively measured physical functioning using reliable and valid measurement instruments.

### Methodological Considerations

The systematic search in the electronic databases was carried out in September 2020, but the search string was not rerun. Therefore, it cannot be excluded that relevant studies published after September 2020 were missed. Moreover, we included only studies from 2000 or later, since we believed that the comparison with IPR studies from before that time are difficult to compare with the IPR of today. Still, we could have missed some important studies that could have altered the results. A strength of this systematic review is that the search strategy was developed with an epidemiologist from the field and a librarian, and we searched in six electronic databases. Furthermore, at least two researchers were involved in all stages and despite the heterogeneity of the selected studies the researchers agreed to the results with a moderate interrater agreement [26]. The discrepancy between the two reviewers involved in the study selection phase made the process more cumbersome, since more studied needed to be discussed with the other researchers involved. Decisions on including- or excluding papers depends on several factors, such as research experience and a clear à priory description of all aspects of PICOTS. However, a prior familiarity with all aspects of PICOTS is difficult to achieve, and this highlighted the need for close communication throughout the entire process, which allowed clarifications to be made. HrQoL is a core outcome in the evaluation of chronic pain treatment [10]. Our outcome was synthesized based on measures with good psychometric properties for chronic pain patients [21–23].

A major difficulty of this study is the heterogeneity of the included studies. First, the heterogeneity is already given by the study population, the different interventions regarding chronic pain, and the heterogeneity for the prognostic factors within the same domain. With the intention of capturing dimensions of the same construct, the grouping of the domains was done with experts from the field and measurement properties that were considered too inaccurate were not included in the analyses [18]. Still, the sources of heterogeneity especially regarding the different statistical analyses prevented the authors from conducting meta-analyses, as the pooling would not be justifiable, and the results would not be interpretable. Since all studies in this review examined different combinations of potential predictors, it was not surprising that conflicting results were shown, for pain intensity, emotional distress or self-reported physical functioning. Moreover, not many studies report the effect sizes of the estimates in order to see if clinical significance was reached, making it difficult to interpret the results beyond statistical significance. In this study, only pretreatment assessments, that is, measures at baseline, were used as prognostic factors to answer the rational of our study, that means that we cannot take into account any influencing factors beyond baseline. However, there are other individual factors that could have affected hrQoL beyond pain rehabilitation (e.g., changes in life circumstances) and should be addressed in future studies. At large, our included studies provided data solely at baseline.

Two-thirds of the studies included in the present review were at high risk of bias and mainly the domain Study Attrition followed by Study Confounding were responsible for this result. Three of the studies were register studies [42–44], which apart from study attrition showed a low overall risk of bias. This raises the question of whether the defined a priori recommendations for the cut-off value of 67% in the Study Attrition Domain for a pain rehabilitation perspective is too restrictive for registry studies or that the classification rules (one high RoB domain classifies the whole study as high RoB) as used previously [28] should be revised. A final possible draw-back of our study is that only studies from Europe, mainly Sweden and Germany, were included in this review and this leads to a questionable generalization of the results for populations from other continents than Europe.

## Conclusion

This systematic review showed low certainty of evidence that cognitive behavioral factors at baseline influence hrQoL at follow-up which is in line with the theoretical approach upon which IPR emanates. For pain factors the results were inconclusive. Moreover, the prognostic factor of initial emotional distress and physical functioning remained unclear. The overall quality of evidence in this review was very low to low, which makes it likely that future studies could impact our findings. To increase the value of prognostic factor investigations of future studies, a predefined core set of predictors investigating hrQoL is needed in patients with chronic pain after IPR. Furthermore, analyzing additional data such as mediating factors and post-intervention outcomes should serve to further contribute to increasing our knowledge in this field.

## Supplementary Data

Supplementary data are available at *Pain Medicine* online.

## Supplementary Material

pnac098_Supplementary_Data

## Notes

Funding sources: No funding has been received for this systematic review.
