# Supplementary material for: Prognostic Factors for Quality of Life After Interdisciplinary Pain Rehabilitation in Patients with Chronic Pain—A Systematic Review
Source: Pain Med. 2022 Jun 23;24(1):52–70. doi: 10.1093/pm/pnac098 (PMC9825145; doi:10.1093/pm/pnac098)
Supplement: pnac098_Supplementary_Data [file pnac098_supplementary_data.zip › pnac098_Supplementary_Data/tableS1.docx]

**Table S1. Search string**

**Medline (Ovid)**

| **#** | **Query** |
| --- | --- |
| 1 | Pain/ |
| 2 | Chronic Pain/ |
| 3 | exp Musculoskeletal Pain/ |
| 4 | exp Back Pain/ |
| 5 | Neck Pain/ |
| 6 | Shoulder Pain/ |
| 7 | Headache/ |
| 8 | Nociceptive Pain/ |
| 9 | Pain, Intractable/ |
| 10 | Myalgia/ |
| 11 | Myofascial Pain Syndromes/ |
| 12 | Fibromyalgia/ |
| 13 | Whiplash Injuries/ |
| 14 | (chronic pain or long-lasting pain or intermittent pain or long-term pain or persistent pain or intractable pain or musculoskeletal pain or chronic muscular pain or nociceptive pain or shoulder pain or neck pain or whiplash or back pain or low back pain or generalized pain or widespread pain or fibromyalgia or myofascial pain syndrome or myalgia or idiopathic pain or diffuse pain or aspecific pain or non-specific pain or musculoskeletal pain syndrome or chronic pain syndrome or somatoform pain syndrome or non-cancer pain or non-malignant pain or benign pain).kf,tw. |
| 15 | 1 or 2 or 3 or 4 or 5 or 6 or 7 or 8 or 9 or 10 or 11 or 12 or 13 or 14 |
| 16 | Pain Management/ |
| 17 | Pain Clinics/ |
| 18 | Rehabilitation/ |
| 19 | Rehabilitation, Vocational/ |
| 20 | rehabilitation.fs. |
| 21 | Rehabilitation Centers/ |
| 22 | Patient Care Team/ |
| 23 | ((multidisciplinary or multiprofessional or multimodal or interprofessional or inter-professional or interdisciplinary or inter-disciplinary or team or biopsychosocial) adj3 (rehabilitation or treatment* or intervention* or regimen* or management or clinic* or therap* or program*)).kf,tw. |
| 24 | functional restoration program.kf,tw. |
| 25 | 16 or 17 or 18 or 19 or 20 or 21 or 22 or 23 or 24 |
| 26 | Forecasting/ |
| 27 | Prognosis/ |
| 28 | exp Probability/ |
| 29 | exp Epidemiologic Factors/ |
| 30 | exp Regression Analysis/ |
| 31 | (predict* or forecasting or prognos* or probability or determinant* or moderator or effect modifi* or Regression Analys* or Logistic Regression or Logistic Model* or Odds Ratio or Risk Ratio or relative risk or risk factor* or protective factor* or Cox model* or Hazard* model* or hazard ratio).kf,tw. |
| 32 | 26 or 27 or 28 or 29 or 30 or 31 |
| 33 | "Quality of Life"/ |
| 34 | Health Status/ |
| 35 | Quality Assurance, Health Care/ |
| 36 | Outcome Assessment, Health Care/ |
| 37 | (health related quality of life or quality of life or SF-36 or short form 36 or SF-12 or short form 12 or 15D or sickness impact profile or Fibromyalgia Impact Questionnaire or the Nottingham health profile).kf,tw. |
| 38 | 33 or 34 or 35 or 36 or 37 |
| 39 | 15 and 25 and 32 and 38 |
| 40 | limit 39 to "review articles" |
| 41 | 39 not 40 |
| 42 | limit 41 to yr="2000 -Current" |

**PsychoINFO (Ovid)**

| **#** | **Query** |
| --- | --- |
| 1 | Pain/ |
| 2 | Chronic Pain/ |
| 3 | exp Back Pain/ |
| 4 | Headache/ |
| 5 | myofascial pain/ |
| 6 | Fibromyalgia/ |
| 7 | Whiplash/ |
| 8 | (chronic pain or long-lasting pain or intermittent pain or long-term pain or persistent pain or intractable pain or musculoskeletal pain or chronic muscular pain or nociceptive pain or shoulder pain or neck pain or whiplash or back pain or low back pain or generalized pain or widespread pain or fibromyalgia or myofascial pain syndrome or myalgia or idiopathic pain or diffuse pain or aspecific pain or non-specific pain or musculoskeletal pain syndrome or chronic pain syndrome or somatoform pain syndrome or non-cancer pain or non-malignant pain or benign pain).tw. |
| 9 | 1 or 2 or 3 or 4 or 5 or 6 or 7 or 8 |
| 10 | Pain Management/ |
| 11 | Rehabilitation/ |
| 12 | Vocational Rehabilitation/ |
| 13 | Rehabilitation Centers/ |
| 14 | ((multidisciplinary or multiprofessional or multimodal or interprofessional or inter-professional or interdisciplinary or inter-disciplinary or team or biopsychosocial) adj3 (rehabilitation or treatment* or intervention* or regimen* or management or clinic* or therap* or program*)).tw. |
| 15 | 10 or 11 or 12 or 13 or 14 |
| 16 | Prediction/ |
| 17 | Prognosis/ |
| 18 | exp Probability/ |
| 19 | exp Statistical Regression/ |
| 20 | (predict* or forecasting or prognos* or probability or determinant* or moderator or effect modifi* or Regression Analys* or Logistic Regression or Logistic Model* or Odds Ratio or Risk Ratio or relative risk or risk factor* or protective factor* or Cox model* or Hazard* model* or hazard ratio).tw. |
| 21 | 16 or 17 or 18 or 19 or 20 |
| 22 | "health related quality of life"/ |
| 23 | "Quality of Life"/ |
| 24 | Health Status/ |
| 25 | (quality of life or SF-36 or short form 36 or SF-12 or short form 12 or 15D or sickness impact profile or Fibromyalgia Impact Questionnaire or the Nottingham health profile or health status).tw. |
| 26 | 22 or 23 or 24 or 25 |
| 27 | 9 and 15 and 21 and 26 |
| 28 | limit 27 to yr="2000 -Current" |

**CINHAL (Ebsco)**

| **#** | **Query** |
| --- | --- |
| S37 | S14 AND S22 AND S29 AND S36 |
| S36 | S30 OR S31 OR S32 OR S33 OR S34 OR S35 |
| S35 | TI ("quality of life" or "SF-36" or "short form 36" or "SF-12" or "short form 12" or "15D" or "sickness impact profile" or "Fibromyalgia Impact Questionnaire" or "the Nottingham health profile" or "health status" ) AND AB ( "quality of life" or "SF-36" or "short form 36" or "SF-12" or "short form 12" or "15D" or "sickness impact profile" or "Fibromyalgia Impact Questionnaire" or "the Nottingham health profile" or "health status" ) |
| S34 | (MH "Sickness Impact Profile") |
| S33 | (MH "Short Form-36 Health Survey (SF-36)") |
| S32 | (MH "Health Status") |
| S31 | (MH "Outcomes (Health Care)") |
| S30 | (MH "Quality of Life") |
| S29 | S23 OR S24 OR S25 OR S26 OR S27 OR S28 |
| S28 | TI ( predict* or forecasting or prognos* or probability or determinant* or moderator or "effect modifi*" or "Regression Analys*" or "Logistic Regression" or "Logistic Model" or "Odds Ratio" or "Risk Ratio" or "relative risk" or "risk factor" or "protective factor" or "Cox model" or "Hazard model" or "hazard ratio" ) AND AB ( predict* or forecasting or prognos* or probability or determinant* or moderator or "effect modifi*" or "Regression Analys*" or "Logistic Regression" or "Logistic Model" or "Odds Ratio" or "Risk Ratio" or "relative risk" or "risk factor" or "protective factor" or "Cox model" or "Hazard model" or "hazard ratio" ) |
| S27 | (MH "Regression+") |
| S26 | (MH "Probability") |
| S25 | (MH "Prognosis") |
| S24 | (MH "Predictive Research") |
| S23 | (MH "Forecasting (Research)") |
| S22 | S15 OR S16 OR S17 OR S18 OR S19 OR S20 OR S21 |
| S21 | TI ( (multidisciplinary OR multiprofessional OR multimodal OR interprofessional OR inter-professional OR interdisciplinary OR inter-disciplinary OR team OR biopsychosocial) N2 (rehabilitation OR treatment* OR intervention* OR regimen* OR management OR clinic* OR therap* OR program*) ) AND AB ( (multidisciplinary OR multiprofessional OR multimodal OR interprofessional OR inter- professional OR interdisciplinary OR inter-disciplinary OR team OR biopsychosocial) N2 (rehabilitation OR treatment* OR intervention* OR regimen* OR management OR clinic* OR therap* OR program*) ) |
| S20 | (MH "Multidisciplinary Care Team") |
| S19 | (MH "Rehabilitation Centers") |
| S18 | (MH "Rehabilitation, Psychosocial") |
| S17 | (MH "Rehabilitation, Vocational") |
| S16 | (MH "Rehabilitation") |
| S15 | (MH "Pain Clinics") |
| S14 | S1 OR S2 OR S3 OR S4 OR S5 OR S6 OR S7 OR S8 OR S9 OR S10 OR S11 OR S12 OR S13 |
| S13 | TI ( ”chronic pain” or ”long-lasting pain” or ”intermittent pain” or ”long-term pain” or ”persistent pain” or ”intractable pain” or ”musculoskeletal pain” or ”chronic muscular pain” or ”nociceptive pain” or ”shoulder pain” or ”neck pain” or ”whiplash” or ”back pain” or ”low back pain” or ”generalized pain” or ”widespread pain” or ”fibromyalgia” or ”myofascial pain syndrome” or ”myalgia” or ”idiopathic pain” or ”diffuse pain” or ”aspecific pain” or ”non-specific pain” or ”musculoskeletal pain syndrome” or ”chronic pain syndrome” or ”somatoform pain syndrome” or ”non-cancer pain” or ”non-malignant pain” or ”benign pain” ) OR AB ( ”chronic pain” or ”long-lasting pain” or ”intermittent pain” or ”long-term pain” or ”persistent pain” or ”intractable pain” or ”musculoskeletal pain” or ”chronic muscular pain” or ”nociceptive pain” or ”shoulder pain” or ”neck pain” or ”whiplash” or ”back pain” or ”low back pain” or ”generalized pain” or ”widespread pain” or ”fibromyalgia” or ”myofascial pain syndrome” or ”myalgia” or ”idiopathic pain” or ”diffuse pain” or ”aspecific pain” or ”non-specific pain” or ”musculoskeletal pain syndrome” or ”chronic pain syndrome” or ”somatoform pain syndrome” or ”non-cancer pain” or ”non- malignant pain” or ”benign pain” ) |
| S12 | (MH "Whiplash Injuries") |
| S11 | (MH "Fibromyalgia") |
| S10 | (MH "Myofascial Pain Syndromes") |
| S9 | (MH "Muscle Pain") |
| S8 | (MH "Nociceptive Pain") |
| S7 | (MH "Headache") |
| S6 | (MH "Shoulder Pain") |
| S5 | (MH "Neck Pain") |
| S4 | (MH "Low Back Pain") |
| S3 | (MH "Back Pain") |
| S2 | (MH "Chronic Pain") |
| S1 | (MH "Pain") |

**Embase (embase.com)**

| **#** | **Query** |
| --- | --- |
| 43 | #42 AND (2000:py OR 2001:py OR 2002:py OR 2003:py OR 2004:py OR 2005:py OR 2006:py OR 2007:py OR 2008:py OR 2009:py OR 2010:py OR 2011:py OR 2012:py OR 2013:py OR 2014:py OR 2015:py OR 2016:py OR 2017:py OR 2018:py OR 2019:py OR 2020:py) |
| 42 | #40 NOT #41 |
| 41 | #40 AND 'review'/it |
| 40 | #16 AND #24 AND #30 AND #39 |
| 39 | #31 OR #32 OR #33 OR #34 OR #35 OR #36 OR #37 OR #38 |
| 38 | 'health related quality of life':ab,ti OR 'quality of life':ab,ti OR 'sf 36':ab,ti OR 'short form 36':ab,ti OR 'sf 12':ab,ti OR 'short form 12':ab,ti OR 15d:ab,ti OR 'sickness impact profile':ab,ti OR 'fibromyalgia impact questionnaire':ab,ti OR 'the nottingham health profile':ab,ti |
| 37 | 'fibromyalgia impact questionnaire'/de |
| 36 | 'health status'/de |
| 35 | 'sickness impact profile'/de |
| 34 | 'nottingham health profile'/de |
| 33 | 'short form 36'/exp |
| 32 | 'quality of life assessment'/de |
| 31 | 'quality of life'/de |
| 30 | #25 OR #26 OR #27 OR #28 OR #29 |
| 29 | predict*:ab,ti OR forecasting:ab,ti OR prognos*:ab,ti OR probability:ab,ti OR determinant*:ab,ti OR moderator:ab,ti OR ((effect NEXT/1 modifi*):ab,ti) OR ((regression NEXT/1 analysis):ab,ti) OR ((logistic NEXT/1 regression):ab,ti) OR ((logistic NEXT/1 model*):ab,ti) OR ((odds NEXT/1 ratio):ab,ti) OR ((risk NEXT/1 ratio):ab,ti) OR ((relative NEXT/1 risk):ab,ti) OR ((risk NEXT/1 factor*):ab,ti) OR ((protective NEXT/1 factor*):ab,ti) OR ((cox NEXT/1 model):ab,ti) OR ((hazard NEXT/1 model):ab,ti) OR ((hazard NEXT/1 ratio):ab,ti) |
| 28 | 'regression analysis'/de |
| 27 | 'epidemiology'/de |
| 26 | 'prognosis'/de |
| 25 | 'prediction'/de |
| 24 | #17 OR #18 OR #19 OR #20 OR #21 OR #22 OR #23 |
| 23 | ((multidisciplinary OR multiprofessional OR multimodal OR interprofessional OR 'inter professional' OR interdisciplinary OR 'inter disciplinary' OR team OR biopsychosocial) NEAR/3 (rehabilitation OR treatment* OR intervention* OR regimen* OR management OR clinic* OR therap* OR program*)):ab,ti |
| 22 | 'rehabilitation center'/de |
| 21 | 'vocational rehabilitation'/de |
| 20 | 'psychosocial rehabilitation'/de |
| 19 | 'functional training'/de |
| 18 | 'rehabilitation'/de |
| 17 | 'pain clinic'/de |
| 16 | #1 OR #2 OR #3 OR #4 OR #5 OR #6 OR #7 OR #8 OR #9 OR #10 OR #11 OR #12 OR #13 OR #14 OR #15 |
| 15 | 'chronic pain':ab,ti OR 'long-lasting pain':ab,ti OR 'intermittent pain':ab,ti OR 'long-term pain':ab,ti OR 'persistent pain':ab,ti OR 'intractable pain':ab,ti OR 'musculoskeletal pain':ab,ti OR 'chronic muscular pain':ab,ti OR 'nociceptive pain':ab,ti OR 'shoulder pain':ab,ti OR 'neck pain':ab,ti OR 'whiplash':ab,ti OR 'back pain':ab,ti OR 'low back pain':ab,ti OR 'generalized pain':ab,ti OR 'widespread pain':ab,ti OR 'fibromyalgia':ab,ti OR 'myofascial pain syndrome':ab,ti OR 'myalgia':ab,ti OR 'idiopathic pain':ab,ti OR 'diffuse pain':ab,ti OR 'aspecific pain':ab,ti OR 'non-specific pain':ab,ti OR 'musculoskeletal pain syndrome':ab,ti OR 'chronic pain syndrome':ab,ti OR 'somatoform pain syndrome':ab,ti OR 'non-cancer pain':ab,ti OR 'non- malignant pain':ab,ti OR 'benign pain':ab,ti |
| 14 | 'whiplash injury'/de |
| 13 | 'fibromyalgia'/de |
| 12 | 'myofascial pain'/de |
| 11 | 'myalgia'/de |
| 10 | 'intractable pain'/de |
| 9 | 'nociceptive pain'/de |
| 8 | 'headache'/de |
| 7 | 'shoulder pain'/de |
| 6 | 'neck pain'/de |
| 5 | 'low back pain'/de |
| 4 | 'backache'/de |
| 3 | 'musculoskeletal pain'/de |
| 2 | 'chronic pain'/de |
| 1 | 'pain'/de |

**Web of Science Core Collection**

| **#** | **Query** |
| --- | --- |
| 7 | #5 not #6 |
| 6 | (#4 AND #3 AND #2 AND #1) AND DOCUMENT TYPES: (Review) |
| 5 | #4 AND #3 AND #2 AND #1 |
| 4 | TS= ("health related quality of life" or "quality of life" or "health status" or "SF-36" or "short form 36" or "SF-12" or "short form 12" or "15D" or "sickness impact profile" or "fibromyalgia impact questionnaire" or "the notthingham health profile") |
| 3 | TS= (predict* or forecasting or prognos* or probability or determinant* or moderator or ”effect modifi” or ”Regression Analys?s” or ”Logistic Regression” or ”Logistic Model” or ”Odds Ratio” or ”Risk Ratio” or ”relative risk” or ”risk factor” or ”protective factor” or ”Cox model” or ”Hazard model” or ”hazard ratio”) |
| 2 | TS= ((multidisciplinary or multiprofessional or multimodal or interprofessional or interdisciplinary or inter-disciplinary or team or biopsychosocial) NEAR/3 (rehabilitation or treatment* or intervention* or regimen* or management or clinic* or therap* or program*) ) |
| 1 | TS= (”chronic pain” or ”long-lasting pain” or ”intermittent pain” or ”long-term pain” or ”persistent pain” or ”intractable pain” or ”musculoskeletal pain” or ”chronic muscular pain” or ”nociceptive pain” or ”shoulder pain” or ”neck pain” or ”whiplash” or ”back pain” or ”low back pain” or ”generalized pain” or ”widespread pain” or ”fibromyalgia” or ”myofascial pain syndrome” or ”myalgia” or ”idiopathic pain” or ”diffuse pain” or ”aspecific pain” or ”non-specific pain” or ”musculoskeletal pain syndrome” or ”chronic pain syndrome” or ”somatoform pain syndrome” or ”non-cancer pain” or ”non-malignant pain” or ”benign pain”) |

**Cochrane Library (Wiley)/ Central**

| **#** | **Query** |
| --- | --- |
| 1 | ("chronic pain" or "long-lasting pain" or "intermittent pain" or "long-term pain" or "persistent pain" or "intractable pain" or "musculoskeletal pain" or "chronic muscular pain" or "nociceptive pain" or "shoulder pain" or "neck pain" or "whiplash" or "back pain" or "low back pain" or "generalized pain" or "widespread pain" or "fibromyalgia" or "myofascial pain syndrome" or "myalgia" or "idiopathic pain" or "diffuse pain" or "aspecific pain" or "non-specific pain" or "musculoskeletal pain syndrome" or "chronic pain syndrome" or "somatoform pain syndrome" or "non-cancer pain" or "non-malignant pain" or "benign pain"):ab,ti |
| 2 | ((multidisciplinary or multiprofessional or multimodal or interprofessional or interprofessional or interdisciplinary or inter-disciplinary or team or biopsychosocial) near/3 (rehabilitation or treatment* or intervention* or regimen* or management or clinic* or therap* or program*)):ab,ti |
| 3 | ("Pain management" or "pain clinic" or "rehabilitation center"):ab,ti |
| 4 | #2 or #3 |
| 5 | (predict* or forecasting or prognos* or probability or determinant* or moderator or "effect modifi*" or "Regression Analys?s" or "Logistic Regression" or "Logistic Model" or "Odds Ratio" or "Risk Ratio" or "relative risk" or "risk factor" or "protective factor" or "Cox model" or "Hazard model" or "hazard ratio"):ab,ti |
| 6 | ("quality of life" or "SF-36" or "short form 36" or "SF-12" or "short form 12" or "15D" or "sickness impact profile" or "Fibromyalgia Impact Questionnaire" or "the Nottingham health profile" or "health status"):ab,ti |
| 7 | #1 and #4 and #5 and #6 with Publication Year from 2000 to 2020, in Trials |
