# Supplementary material for: Prognostic Factors for Quality of Life After Interdisciplinary Pain Rehabilitation in Patients with Chronic Pain—A Systematic Review
Source: Pain Med. 2022 Jun 23;24(1):52–70. doi: 10.1093/pm/pnac098 (PMC9825145; doi:10.1093/pm/pnac098)
Supplement: pnac098_Supplementary_Data [file pnac098_supplementary_data.zip › pnac098_Supplementary_Data/tableS2.docx]

**Table S2. Detailed Outcome and Analyses**

| **Author (year)** | **Analysis** | **Dependent variables** | **Independent variables (adjusted for)** | **Details** |
| --- | --- | --- | --- | --- |
| Angst et al. (2014) | regression analyses | SF-36  subscale  PF: score 0-100  used as a continuous variable | potential confounders controlled for in regression analysis:  sex, age, education, partnership, number of comorbidities, BMI, sports, smoking, medication with analgesics/antidepressants, type of accident, disease duration, CSQ activity level  further independent co-variates (included as predictors to test the hypothesis):  SF-36 PF, SF-36 bodily pain, HADS-D, HADS-A, CSQ control pain, CSQ decrease pain, CSQ catastrophizing | All covariates were tested by stepwise inclusion. Covariates with a bivariate correlation to the dependent variable with a significance of p-value <0.300 were included in the model: the one with the highest bivariate correlation as first, the one with the second highest bivariate correlation as second etc.  If the multivariate correlation of that variable had a significance of p-value <0.100 (by the F-test) it was included in the final model. |
| Bremander et al. (2011) | multivariate logistic regression | SF-36  subscales  PF, MH: score 0-100  outcome dichotomized improved/nonimproved | gender, age, antidepressant medication, VAS pain, pain distribution, anxiety and depression | Each of the candidate variables was tested separately in multivariate analyses controlling for age and gender. |
| Buchner et al. (2007) | ANOVA and Kruskal-Wallis test | SF-36  summary scales  PCS, MCS: score 0-100  used as a continuous variable | grade of chronicity  - Group A: Stage I and II  - Group B: Stage III  - Group C: Stage VI | All values were expressed as means for continuous data or as counts and percentages for discrete data. A p-value of ≤0.05 was considered to be statistically significant. |
| Dong et al. (2019) | multivariate linear mixed regression analyses | SF-36  summary scales  PCS, MCS: score 0-100  used as a continuous variable | Model 3:  BMI group, change in BMI, time and its interaction with BMI, university/college, sex, work/study, born in Sweden, NRS-7d, PRI, HADS-A, HADS-D | Model 1: only BMI group, change in BMI, time and its interaction with BMI group were tested  Model 2: sociodemographic characteristics were added  Model 3: pain aspects and psychological symptoms were added |
| Farin et al. (2013) | multivariate regression analyses  with multiple imputation | SF-12  subscales  PCS, MCS: score 0-100  used as a continuous variable | SF-12 PCS:  age, income, comorbidity score, pain intensity, FABQ-work beliefs, patient-physician relationship variables  SF-12 MC:  treatment motivation, FABQ-work beliefs, fatalistic external locus of control, IPQR: coherence, patient–physician relationship variables | Step 1: adjustment for center effects  Step 2: all confounding sociodemographic variables were added  Step 3: medical variables were added  Step 4: psychological variables were added  Step 5: the characteristics of physician–patient relationship were included  All variables were added as fixed-effect coefficients in the level 2 model.  Predictors which proved to be significant in at least two of the five imputed data sets were considered as potentially relevant predictors.  Finally, the models that consisted of only potentially relevant predictors were again applied to all five imputed sets. |
| Gerdle et al. (2016) | multivariate regression analyses with PLS | SF-36  summary scales  PCS and  MPI Pain Interference factorized into one outcome  used as a continuous variable | NRS-7days, MPI-Pain severity, MPI-pain interference, SF-36 PF, HADS-D, HADS-A, MPI-distress, MPI-LifeControl, CPAQ, SF-36 PCS, Question RTW-when and own prognosis RTW | The PLS analyses were made in two steps.  Step 1: all potential regressor variables were entered  Step 2: If this regression was significant, regressors with VIP >0.80 were selected and used in the final PLS |
| Glattacker et al. (2010) | hierarchical multiple regression analyses | SF-36  subscales  PF, MH: score 0-100  used as a continuous variable | age, level of education, employment status, partnership, period of time since diagnosis, the baseline health status, self-efficacy, illness representations | Step 1: baseline health status was entered into the regression  Step 2: socio-demographic variables, period of time since diagnosis, as well as general self-efficacy were entered  Step 3: illness representations were entered |
| Glattacker (2018) | multivariate linear regression analyses | SF-12  summary scales  PCS, MCS: score 0-100  used as a continuous variable | IPQR: 8 dimensions, FABQ, CPQ, PCAS, HADS, PSEQ, age, sex, baseline score (SF-12 PCS, SF-12 MCS), pain intensity | Stage 1: assessed the contribution of each of the individual psychological constructs in predicting outcomes after adjusting for baseline scores of the outcome variables, demographic variables and baseline pain intensity in separate models  Model 1: included the baseline scores of the outcome variables  Model 2: added demographic variables and baseline pain intensity  Model 3: added one psychological construct  Stage 2: The multivariate models included those multiple psychological constructs that were found to be statistically significant at Stage 1. The models controlled for the baseline outcome scores, age, sex and baseline pain intensity. |
| Heiskanen et al. (2012) | ANOVA and Pearson’s chi- square test | 15D  used as a continuous variable | pain duration  pain intensity  compared but not in the same analysis:  age, sex, marital status, education level, type of pain | For continuous variables: ANOVA for the significance of the differences between the groups followed by post hoc comparisons with independent samples t-test.  For categorical variables: Pearson’s chi-square test; p-values <0.05 were considered as statistically significant. |
| Martin et al. (2014) | multivariate regression analyses | FIQ  one global score 1-100  used as a continuous variable | univariate analysis:  age, sex, marital status, level of education, employment status, physical illnesses, number of years since the onset of pain, number of tender points, CAD-R, FIQ  multivariate analysis:  FIQ, numer of physical illnesses, tender points, CAD-R | A univariate analysis was performed initially and variables with p-value <0.20 were included in the multivariate analysis. |
| Martin et al. (2017) | multivariate linear mixed model | FIQ  one global score 1-100  used as a continuous variable | patients’ clinical and sociodemographic characteristics  gender, age, marital status, educational level, employment status, tender points, number of concurrent illnesses, years since the onset of pain, HADS-A, HADS-D  final model:  marital status, number of concurrent physical illnesses, years since onset of pain, HADS-A | To assess the unadjusted effects of the covariates on the main outcome, generalized linear mixed models were used.  Patient characteristics with p values <0.20 were introduced into the multivariate model. |
| Moradi et al. (2010) | ANOVA and Kruskal–Wallis | SF-36  subscales  PF, MH: score 0-100  used as a continuous variable | pain site:  - single-site  - dual-site  - multiple-site  covariate analyses regarding educational level and smoking | ANOVA test for quantitative variables and Kruskal–Wallis test for categorical variables among the three groups were performed at follow-up. |
| Orenius et al. (2013) | multivariate logistic regression analyses | 15D  Outcome dichotomized, cutoff is MCID: ≥0.03 on the 0–1 scale | age, sex, BDI, BAI, TSK | Multivariate logistic regression model was used to estimate odds ratios and their 95% intervals when predicting 15D change. |
| Tseli et al. (2020) | multiple logistic regression analyses | SF-36  summary scales  PCS: 0-100  MCS combined with HAD: 0-100  outcome dichotomized, cut-off is MCID: ≥3 points SF-36 and ≥1.5 HAD | Final model:  PCS:  age, employment status, belief of restored health, number of pain regions, NRS 7-d, MPI pain interference, MPI overall activity, HADS-A, SF-36 MCS, SF-36 PCS  MCS/HAD:  sex, education level, country of origin, employment status, NRS-7d, MPI life control, MPI overall activity, SF-36 MCS | The final multiple regression model was identified in a 2-step procedure.  Step 1: stepwise backwards elimination was used to eliminate variables based on the highest p-value, until only variables significant at p ≤0.2 remained.  Step 2: variables already eliminated in the univariate analyses were included one by one in the multiple regression model and retained if they were significant at p <0.05, yielding a preliminary final model with all variables significant at p ≤0.2. Baseline regressors significant at p <0.05 in the final model were recognized as important prognostic factors. |

Legend: ANOVA: ANalysis Of Variance; BAI/BDI: Beck Anxiety and Depression Inventory; BMI: Body Mass Index; CAD-R: Coping with Chronic Pain Questionnaire; CPAQ: Chronic Pain Acceptance Questionnaire; CPQ: Coping Procedures Questionnaire; CSQ: Coping Strategies Questionnaire; FABQ: Fear Avoidance Belief Questionnarie; FIQ: Fibromyalgia Impact Questionnaire; HADS: Hospital Anxiety and Depression Scale; IPQR: Illness Perception Questionnaire; MCID: Minimal Clinical Important Difference; MCS: Mental Component Summary; MH: Mental Health; MPI: Multidimensional Pain Inventory; NRS-7d: Numeric Rating Scale 7 days; PCS: Physical Component Summary; PCAS: Pain Catastrophizing Scale; PH: Physical Health; PLS: Partial Least Square regression; PSEQ: Pain Self-Efficacy Questionnaire; PRI: Pain Region Index; RTW-expect: expectation of Return To Work: RTW-prognosis: perceptions of prognosis on Return To Work; SF-36/12: 36/12-Item Short Form Health Survey; VAS: Visual Analog Scale; VIP: Variable Influence on Projection; TSK: Tampa Scale of Kinesiophobia; 15D: 15-dimensional health-related quality of life
